# The Year 2022 in Cardiovascular Diseases—*Clinics and Practice*

**DOI:** 10.3390/clinpract13040069

**Published:** 2023-07-05

**Authors:** Maurizio G. Abrignani

**Affiliations:** Operative Unit of Cardiology, P. Borsellino Hospital, Marsala, ASP Trapani, 91016 Erice, Italy; maur.abri60@gmail.com

This journal has recently achieved significant milestones, receiving its first impact factor of 2.3 and CiteScore of 2.0. *Clinics and Practice* is especially devoted to the clinical aspects of medicine, not disregarding basic and translational research. The interests of this journal, consequently, cover a wide spectrum of topics, focusing on pain, sport, reproductive medicine, infectious diseases, oncology, pediatrics, dentistry, psychology, psychiatry, clinical pharmacology, public health, and intensive care [1]. Of course, publications on cardiovascular disease, the main cause responsible for mortality and morbidity throughout the world, represent another important aspect of this evolving journal. In the last year 2022, some important papers on this field have been published in *Clinics and Practice*, and this editorial aims to briefly summarize them.

The year 2022 will be remembered as an exceptional year for re-establishing a near-normal life after the deadly pandemic that affected millions of people worldwide. However, Coronavirus Disease-19 (COVID-19) still represents an important field for discussion in scientific literature. SARS-CoV-2 infection was shown to be frequently associated with cardiovascular complications, including venous and arterial thromboembolic events [2]. Viral spike proteins, in fact, may promote the release of prothrombotic and inflammatory mediators. COVID-19-related venous thromboembolism (VTE) is associated with increased mortality. Several global guidelines recommended prophylactic-intensity anticoagulation rather than intermediate-intensity or therapeutic-intensity anticoagulation for patients with COVID-19-related acute or critical illness without suspected or confirmed VTE [3], but many cases of thrombotic complications have been reported, even though standard doses of thromboprophylaxis are received. Hence, appropriate and adequate thromboprophylaxis is critical for VTE prevention in COVID-19. In this journal, Ramakrishnan et al. [4] reviewed the available global literature, providing clinical insights into our approach towards managing VTE in patients with COVID-19. Furthermore, they summarized the incidence and risk factors for VTE with an emphasis on the thromboprophylaxis approach in hospitalized patients and special populations with COVID-19, assessing clinical implications in their Indian context.

Vaccines coding for the spike protein, are the primary means of preventing COVID-19 morbidity and mortality. However, some unexpected thrombotic events at unusual sites, most frequently located in the cerebral venous sinus, but also in the splanchnic district, with associated thrombocytopenia, have emerged in subjects who received adenovirus-based vaccines, especially in fertile women [2]. This clinical entity was soon recognized as a new syndrome, named vaccine-induced immune thrombotic thrombocytopenia, probably caused by cross-reacting anti-platelet factor-4 antibodies activating platelets. For this reason, the regulatory agencies of various countries restricted the use of adenovirus-based vaccines to some age groups. The prevailing opinion of most experts, however, is that the risk of developing COVID-19, including its thrombotic complications, clearly outweighs this potential risk, and thus vaccines offer a net positive effect. Also, in *Clinics and Practice*, Sessa et al. [5] reported a clinical case of a thrombotic event in an unusual site (ulnar artery) in the same side of the body where the COVID-19 vaccination took place. The patient (69-year-old male) had no changes after a laboratory investigation regarding thrombophilic pattern, but nevertheless he had atherothrombotic predisposing conditions.

Ayo Bivigou et al. [6] analyzed the effect of hydroxychloroquine or chloroquine associated with azithromycin on the QTc interval in 224 patients treated for COVID-19 at the Libreville University Hospital Center in Gabon. After 48 h of treatment, 50 (22.3%) patients had a significant prolongation of QTc. This tended to be more frequent in patients treated with chloroquine (*n* = 33; 27.0%) than in those treated with hydroxychloroquine (*n* = 17; 16.7%) (*p* = 0.06). QTc prolongation exceeding 60 ms was found in 48 (21.3%) patients, while 11 patients had a (4.9%) QTc exceeding 60 ms at admission and exceeding 500 ms after 48 h. Thus, the authors confirmed that early QTc prolongation was frequent in COVID-19 patients treated with hydroxychloroquine or chloroquine in association with azithromycin [7].

Two interesting papers have been published in this journal in the field of cardiovascular prevention.

The treatment of high blood pressure is a combination of lifestyle changes and medications, and appropriate exercise therapy is recommended as one of the lifestyle-related changes. Recently, stretching, a low-intensity exercise, was reported to be antihypertensive and effective for improving arteriosclerosis, in addition to aerobic exercise [8]. Yamada et al. [9] investigated the short-term effects of continuous stretching and rest-induced rebound on vascular endothelial function in ten hypertensive patients between 30 and 70 years of age. The intervention consisted of six months of daily stretching, one month of rest, and another three months of stretching. They measured arteriosclerosis indices such as cardio ankle vascular index (CAVI), ankle brachial pressure index (ABI), and reactive hyperemia index (RHI), and flexibility at the baseline and after one, three, six, seven, and ten months. The anteflexion measurement value improved significantly before and after the intervention (*p* < 0.001). Blood pressure and CAVI/ABI were well controlled throughout the study period. RHI did not show any significant improvement during the initial six months, and only slightly improved by the third month (*p* = 0.063). Even after the rest phase and the resumption of stretching, RHI remained stable. Thus, in this study the authors did not observe a significant positive effect on arteriosclerosis index or blood pressure, although the compliance with this stretching program, evaluated by the exercise implementation rate for the entire period, was 90% or more. This is likely to be due to the short-term intervention period.

The lipid-lowering landscape is rapidly evolving. With the increasing global burden of dyslipidemia over the past 30 years, it is estimated that more than 200 million people worldwide are under statin therapy. Although statins, on the one hand, are highly effective against dyslipidemia and cardiovascular diseases, on the other hand they may cause adverse effects, including an increased risk of diabetes mellitus. Needamangalam Balaji et al. [10] conducted a scoping review on 48 published articles selected from PubMed and GoogleScholar. Their results confirmed that statin users are potentially at greater risk of developing diabetes mellitus compared with patients who do not use statins [11]. The exact mechanism is not yet precisely established, and thus future studies are essential for identifying the cause of diabetes mellitus in statin users.

The year 2022 has been a notable year in heart failure (HF), and *Clinics and Practice* published three studies on this condition. Sodium–glucose co-transporter 2 (SGLT2) inhibitors are becoming one of the main treatments for patients with cardiorenal disease [12]. Empagliflozin, a SGLT2 inhibitor, has been shown to bind to late sodium channels in mice cardiomyocytes. Antwi-Amoabeng et al. [13] sought to investigate the electrocardiographic features associated with empagliflozin use, finding that empagliflozin was associated with a significant increase in QRS duration among diabetes patients with heart failure.

Effective HF treatment involves care with food intake [14]. Recently, the Brazilian Cardioprotective Diet and its dietary index, BALANCE, which assesses adherence to the standard’s recommendations, was proposed by the local Ministry of Health. In an observational prospective study, part of the Congestive Heart Failure Registry (VICTIM-CHF), Costa et al. [15], studying 240 patients with HF, showed that individuals with a partner have a greater adherence to the green food group recommendations, whereas the lowest adherence to recommendations regarding the blue food group was observed in individuals with excess weight, who had a higher consumption of foods rich in animal protein. As for the red food group (ultra-processed foods), the highest adherence was observed by patients with diabetes mellitus. The greatest adherence to the yellow food group, and a higher score, was observed in patients with the smallest left ventricular systolic diameter.

Cardiac Contractility Modulation has been proposed for inpatients affected by reduced ejection fraction HF, with relapsing symptoms [16]. Matteucci et al. [17] presented a case of a patient treated with percutaneous coronary intervention in the setting of acute coronary syndrome (ACS) without persistent ST-segment elevation, with the best medical therapy for decompensated HF. The patient refused the implantable cardioverter-defibrillator, and to reduce the increasing number of hospitalizations for HF exacerbations they proposed instead the use of a cardiac contractility modulation device. After the implant, the patient demonstrated a marked improvement in exercise effort and quality of life (QOL), assessed with a six-minute walk test, the Minnesota Living with Heart Failure Questionnaire, and echocardiographic parameters. At 9 months after discharge, no hospital admissions for HF were recorded. The authors showed how the improvement in global longitudinal strain correlates with the remodeling effects on myocardial cells using speckle tracking imaging.

Pulmonary embolism (PE), a frequently underdiagnosed and potentially fatal condition, is the third most common vascular disease in the US. Interventional and surgical treatments are available nowadays [18]. Raghupathy et al. [19], in this journal, described the prevalence, outcomes, and predictors of mortality of PE patients treated with mechanical (MT) and surgical thrombectomy (ST) using the Agency for Healthcare Research and Quality’s HCUPNIS data. The utilization trend of MT increased from 336 (0.20%) in 2010 to 1655 (0.87%) in 2018; the utilization trend of ST was 260 (0.15%) in 2010 and 430 (0.23%) in 2018. The unadjusted in-hospital mortality for MT was 9.1%, with the mean length of stay (LOS) being 7 (±0.3) days; for ST, mortality was 13.9% with a mean LOS of 13 (±0.4) days. The occurrences of periprocedural complications for MT and ST were, respectively, invasive mechanical ventilation (13.8% and 32%), cardiopulmonary bypass (3.3% and 68.3%), and bleeding complications (1.4% and 3.4%). Predictors associated with in-hospital mortality for MT were increasing age, female sex, large hospitals, and teaching hospitals. The predictor of in-hospital mortality for ST was increasing age.

In the field of valvular heart disease, bioprosthetic valve thrombosis is considered a relatively rare but life-threatening clinical entity [20]. Thus, there is the need for high clinical suspicion to make a timely diagnosis and related appropriate therapeutic interventions. In this regard, the management of this condition is at high risk, whatever the option taken (surgery and/or systemic fibrinolysis). The presence of severe comorbidities—as decompensated cirrhosis—further complicates the clinical decision-making process, calling for a patient-tailored integrated multidisciplinary approach. Cocchia et al. [21] reported here a challenging case of a 45-year-old patient with mitral bioprosthetic valve thrombosis and hepatitis C virus-related cirrhosis complicated by active duodenal variceal bleeding.

QOL is used as a health indicator to assess the effectiveness and impact of therapies in certain groups of patients [22]. In *Clinics and Practice*, de Carvalho Costa et al. [23] aimed to analyze the QOL of patients with ACS who received medical treatment from a public or private health care system in an observational, prospective, longitudinal study carried out in Sergipe, Brazil. At 180 days after ACS, the public health care group had lower QoL scores for all domains (functional capacity, physical aspects, pain, general health status, vitality, social condition, emotional profile, and health) (*p* < 0.05) than the private group. The highest QoL level was associated with male sex (*p* < 0.05) and adherence to physical activity (*p* ≤ 0.003) for all assessed domains. Thus, social factors and health status disparities influence QoL after ACS.

New-onset atrial fibrillation (NOAF) is one of the leading causes of morbidity and mortality, especially in older patients in the intensive care unit [24]. Many comorbidities are associated with NOAF. Sertcakacilar et al. [25], in this journal, tested the hypothesis that anemia is associated with an increased risk of developing NOAF in critically ill patients. Their analysis of 1931 patients actually revealed that anemia is associated with the development of NOAF in critically ill patients in intensive care.

Finally, two studies on vascular medicine were published in *Clinics and Practice* in 2022. Uterine artery pseudoaneurysm is a rare and potentially life-threatening vascular anomaly caused by the inadequate sealing of a ruptured wall of a uterine artery, mainly occurring after a traumatic lesion [26]. It can lead to delayed postpartum hemorrhage. Böckenhoff et al. [27] reported a rare case of this condition after an uncomplicated vaginal delivery in a patient with a history of deep-infiltrating endometriosis. Selective coil embolization was successfully performed. They concluded that uterine artery pseudoaneurysm should always be considered in cases of unexplained abdominal pain after surgery or childbirth with or without vaginal bleeding. Lungu et al. [28] published a case report of a 5.6 cm glomangioma supplied by the femoral profunda artery in a 66-year-old male patient with severe peripheral artery disease. The patient complained of discomfort and mild pain at the place of the lesion and an accelerated growth rate in the last two months. A nodular mass located laterally on the left foot, elastic, covered with a thin skin, and mobile, was noted on the clinical exam. A Doppler exam demonstrated an active vascular supply. CT angiography showed femoral profunda artery blood supply and severe asymptomatic bilateral peripheral artery disease. The lesion was removed entirely by surgery. A microscopy exam revealed a glomangioma. After surgery, the patient recovered unevenly.

I expect that this journal will continue publishing other equally interesting, quality papers in the field of cardiovascular diseases in 2023.
